# Nanodrug delivery system constructed with dopamine-based functional molecules for efficient targeting of tumour cells

**DOI:** 10.1039/d4ra05654j

**Published:** 2024-09-30

**Authors:** Zhifeng Chen, Jinglun Dai, Nian Fu, Zhihong Yan, Qinfang Zheng, Yi Liu

**Affiliations:** a School of Chemistry and Chemical Engineering GDPU, Guangdong Pharmaceutical University China wdgzyx321@163.com liuyi_papers@163.com; b Key Laboratory of Dong Medical Research of Hunan Province, Hunan University of Medicine China; c School of Pharmacy GDPU, Guangdong Pharmaceutical University China; d School of Traditional Chinese Medicine GDPU, Guangdong Pharmaceutical University China; e Guangdong B. C. Biotech Co., Ltd China

## Abstract

In order to enhance the water solubility of chemotherapeutic drugs, improve their biodistribution and narrow therapeutic window, two molecules of PDAO and FA-DAO with acid-sensitive Schiff base structure were designed and synthesized based on dopamine linolenate (DAO) in this paper, and subsequently drug-loaded nanoparticles were prepared by simply mixing of them with curcumin (Cur) in aqueous solution. These nanoparticles can release a large amount of the drug in response to pH changes in the tumor microenvironment through passive targeting. The cumulative rate of drug release can reach up to 70% within 24 hours under pH = 5.0 conditions as a release medium. Furthermore, the drug-carrying nanoparticles achieve active targeting through folic acid (FA) on their surface, which further enhances targeting efficiency. The inhibitory effect of drug-loaded nanoparticles was nearly 8-fold enhanced than that of its loaded Active Pharmaceutical Ingredient (API) Cur on HepG2 cell lines at the administration concentration of 6.25 μg mL^−1^. In conclusion, the nanoparticles prepared in this work improved the aqueous solubility of the loaded drug Cur, where passive targeting provided by pH-responsiveness and active targeting provided by FA endowed the loaded drug Cur with highly efficient targeting of HepG2 cell lines.

## Abstract 2

This work used three types of molecules to construct targeted nanoparticles that can enhance the anti-tumor effect of Cur through active and passive targeting used three moleculars to construct targeted nano.

## Graphical abstract

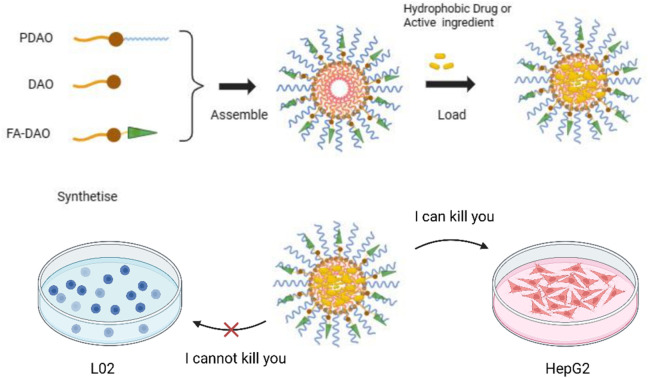

## Introduction

Chemotherapy is a very effective method of tumor prevention and treatment,^1^ however, common problems such as poor solubility of chemotherapeutic drugs, rapid blood clearance, and wide biodistribution^2^ have led to these chemotherapeutic drugs having high toxicity and side effects. Therefore, how to improve the drug solubility and deliver the drugs to the targeted tissues and cells, and improve their biodistribution, has become an urgent problem to be solved.

Nanomedicine and nano-delivery systems are a science that has gradually developed in recent years. It involves specific materials at the nanoscale.^3^ Through the rational design of nano-delivery systems, drug solubility can be effectively improved. Qizhou Chen *et al.*^4^ synthesized a new type of amphiphilic chitosan wall material to effectively load curcumin (Cur), Alaa K. Othman *et al.*^5^ coated polydiallyldimethylammonium chloride (PDDA) and silica nanoparticles on the surface of liposomes by layer-by-layer assembly method, and completely encapsulated Cur in the core of liposomes to achieve efficient loading of Cur. Although these nanoparticles can well load drugs, the large particle size and lack of targeting ability cannot further exert the anti-cancer effect of drugs.

Due to the Warburg effect,^6^ tumor cells produce a large amount of lactic acid in anaerobic glycolysis metabolism, making the pH of the tumor microenvironment lower than that of normal tissues. Therefore, the designed acidity-sensitive drug delivery system can enable the drug to accumulate at the tumor site to achieve passive targeting. Schiff base^7^ is formed by condensation of amines and active carbonyl groups. Its structure is R–C=N–R, which is easily broken under acidic conditions to form corresponding bases and amines. Guoling Li^8^ used this structure to connect doxorubicin (DOX) with terephthalaldehyde and amino-containing mesoporous nano-silica to prepare a pH-responsive drug delivery system to achieve specific release of tumor cells. Zhihang Yang *et al.*^9^ prepared nanoparticles with Schiff base structure by connecting bovine serum albumin nanoparticles and DOX through glutaraldehyde. Their *in vitro* drug release experiments showed that acidity significantly accelerated the release of DOX due to the decomposition of nanoparticles (NPs) induced by the cleavage of Schiff base bonds. This passive targeting improves the efficacy of the drug to a certain extent.

Dopamine (DA) is often made into polydopamine (PDA) in drug delivery systems and used as a substrate or coating to prepare nanoparticles, which are widely used due to their high biocompatibility.^10^ At the same time, dopamine contains aromatic rings, which can achieve drug loading through π–π stacking and hydrogen bond interaction.^11^ In addition, dopamine contains catechol reactive groups, which can promote its covalent binding to compounds containing amino or thiol groups through Michael addition or Schiff base reaction^12^ to achieve functionalization. Yang Lu *et al.*^13^ synthesized polydopamine-loaded paclitaxel to prepare anti-tumor nanoparticles for drug combined with photothermal therapy. Haijun Wang *et al.*^14^ developed Cur-loaded porous (poly lactic acid-*co*-glycolic acid) (PLGA) nanoparticles (NPs) by nanoprecipitation method, which showed longer blood circulation time and excellent photothermal properties, allowing tumor-specific chemical photothermal therapy. Zeyu Li *et al.*^15^ demonstrated a multifunctional nanoparticle with imaging-guided chemo-photothermal synergistic therapy and targeted delivery of EpCAM antibody to liver tumor cells. They linked the active group amino group on the surface of polydopamine to the antibody through amidation reaction to make the nanoparticles have active targeting ability. Although these polydopamine-coated nanoparticles can be combined with photothermal therapy or modified to have certain advantages in tumor therapy, the formation of dopamine coating requires control of time, pH, *etc.* The preparation process is complex, and the limited targeting ability limits the wide application of this polydopamine nanoparticle.

Therefore, this study proposes to synthesize multifunctional molecules PDAO and FA-DAO, and then based on the preparation method of mixed micelles, these molecules are compounded to make nanoparticles with active-passive targeting. Specifically, firstly, DA and linolenic acid were linked by amidation reaction to synthesize relatively hydrophobic linolenic acid dopamine (DAO), and then a folic acid (FA)-modified molecule FA-DAO for tumor cell targeting and a PEG-modified amphiphilic molecule PDAO with aromatic ring parent nucleus were designed by using Schiff base structure. The nano-delivery system prepared with the above three molecules does not require a complicated preparation process. They can spontaneously form drug-loaded nanoparticles with small particle size, well pH response and efficient tumor cell targeting in aqueous solution by mixing them with drugs.

## Result and discussion

### Analysis of infrared spectra and ^1^H-NMR spectrum of the PDAO, DAO and FA-DAO

**Fig. 1:**
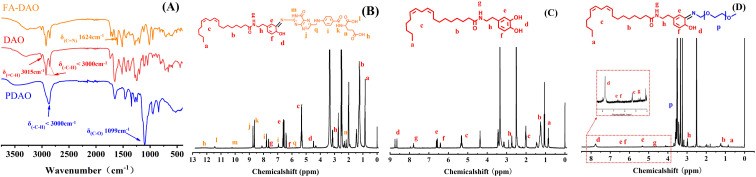
(A) Infrared spectra of PDAO, DAO and FA-DAO. NMR hydrogen spectra of (B) FA-DAO, (C) DAO and (D) PDAO.

The IR spectra of the three products synthesised are shown in Fig. 1(A), and the characteristic peaks of 1500–1700 cm^−1^ of the aromatic ring attributed to the DA backbone are observed in the IR profile of the product DAO, while unsaturated hydrocarbon stretching vibration peaks at 3015 cm^−1^ and less than 3000 cm^−1^ and saturated hydrocarbon stretching vibration peaks can also be observed. PDAO and FA-DAO were synthesised from DAO, so that the characteristic peak corresponding to 1099 cm^−1^ could be observed in the infrared spectrum of PDAO. When PEG was introduced on DAO, it led to a significant increase in the saturated hydrocarbon stretching vibration peaks and C–O stretching vibration peaks less than 3000 cm^−1^ in the product. FA was introduced on DAO for the synthesis of FA-DAO and the attributed C=N stretching vibration peak at 1624 cm^−1^ could be observed in its profile.^16^

The ^1^H-NMR profiles of the three products synthesised, as shown in Fig. 1(B), show that the DAO hydrogen spectrum is fully attributed, and the pro-diphenol hydroxyl group is the peak at d, which changes to the keto form of dopamine quinone under alkaline conditions,^17^ with which the mPEG-NH_2_ and FA can react *via* the amino group to form the Schiff base structure. In Fig. 1(D) PDAO, the original double peak at d becomes a single peak, and the chemical shift decreases due to the change of substituent, implying the successful introduction of a new substituent and the formation of the Schiff base structure, and the distinct characteristic peak of hydrogen in PEG at 3.5 ppm proves that PEG is successfully connected to DAO through the Schiff base structure; in figure FA-DAO, the peaks at 8.5–9 ppm are attributed to the peaks at j and k of hydrogen in FA,^18^ the peaks of m, l, and h are attributed to the peaks of hydrogen on the two carboxyl groups in FA and the peak of hydrogen on the pteridin parent nucleus, respectively, and the peak of phenolic hydroxyl group is chemically shifted reduced to 4.3 ppm due to the introduction of FA. The above results demonstrate the successful synthesis of FA-DAO, PDAO and DAO.

### Observation of nanoparticle size and its morphology under TEM

**Fig. 2:**
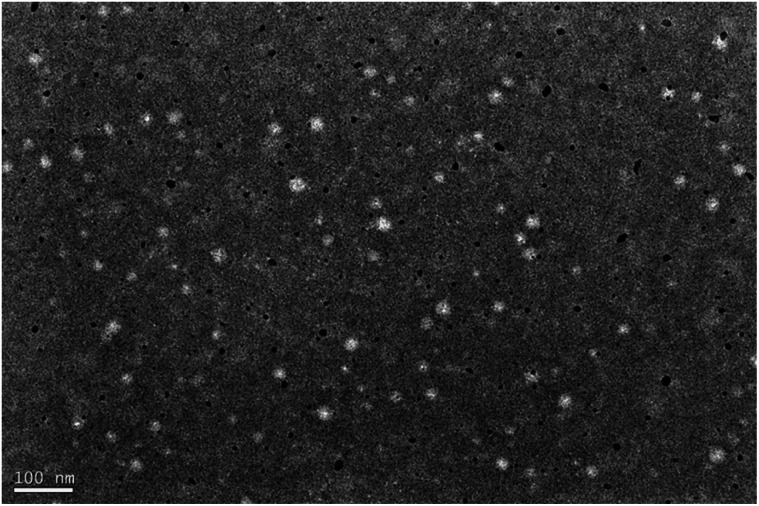
TEM image of nanoparticles with PDAO, DAO and FA-DAO = 1.0 : 0.3 : 0.1.

After pre-experimentation the ratio of PDAO and DAO was determined to be 1.0 : 0.3, as shown in the Table 1, the composite nanoparticle size gradually increased with the increase of FA-DAO content, this is because FA-DAO in this nanoparticle is hydrophobic and will be wrapped by the nanoparticle in the nanoparticle hydrophobic portion, and with the increase of FA-DAO content, hydrophobic core is also increased,^19^ so the nanoparticle particle size becomes larger. The nanoparticles prepared in the ratio of PDAO, DAO and FA-DAO = 1.0 : 0.3 : 0.1 have smaller particle size, which is favourable for biodistribution. From the TEM image, it can be seen that the nanoparticles are regular spherical, uniform in size, no adhesion phenomenon, and the particle size is around 50 nm, which is smaller than the hydrated particle size, whereas its whereas the particle size measured by Malvern laser particle sizer is around 150–200 nm, which is due to the fact that the particle size measured by the TEM is the dried and dehydrated particle size, and the Malvern laser particle sizer is the particle size of this nanoparticle in aqueous solution is the hydrated particle size^20^ (Fig. 2).

**Table 1:** Relationship between nanoparticles and particle size for different ratios of PDAO, DAO, FA-DAO

PDAO : DAO : FA-DAO	Size (nm)	PDI
1.0 : 0.3 : 0.1	146.53 ± 16.50	0.362
1.0 : 0.3 : 0.2	188.97 ± 7.40	0.305
1.0 : 0.3 : 0.3	170.53 ± 2.60	0.311
1.0 : 0.3 : 0.4	190.90 ± 15.30	0.348
1.0 : 0.3 : 0.5	224.00 ± 2.70	0.251

### Critical aggregation concentrations (CAC) and its formation mechanism of nanoparticles

**Fig. 3:**
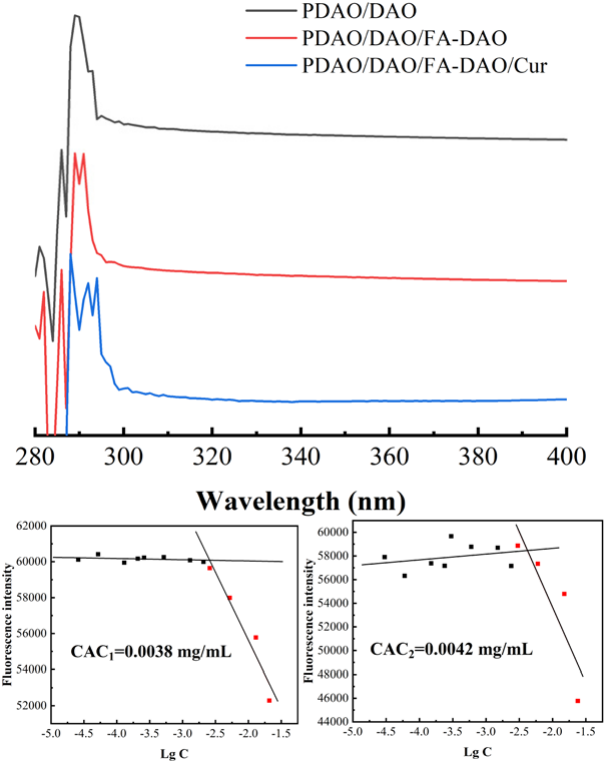
UV of different nanoparticle, CAC1 with PDAO : DAO = 1 : 0.3 and CAC2 with PDAO : DAO : FA-DAO = 1 : 0.3 : 0.1.

PDAO is an amphiphilic molecule. The hydrogen bond provided by the PEG part and the hydrophobic interaction force provided by the oleic acid part are the main driving forces to promote the formation of nanoparticles. The critical aggregation concentrations (CAC) of nanoparticles was determined by pyrene fluorescence method. And the CAC of the composite nanoparticles were 0.0038 mg mL^−1^ for CAC1 and 0.0042 mg mL^−1^ for CAC2 with the sequential incorporation of DAO and FA-DAO, respectively, and their CAC is small, which may be due to the fact that the FA fragments and DAO in FA-DAO enhanced the structure of micelles by π–π stacking.^21,22^ This phenomenon can also be observed in the ultraviolet spectrum Fig. 3, the 290 nm wavelength absorption peak generated by the π → π* transition is redshifted because of the increase of aromatic rings in the nanoparticles, which causes the different molecules to aggregate and stack through π–π interaction.^23,24^ In addition, the lower critical aggregation concentration allowed the nanoparticles to possess better resistance to dilution and drug solubilisation.^25^

### Effect of pH on the particle size of nanoparticles

**Fig. 4:**
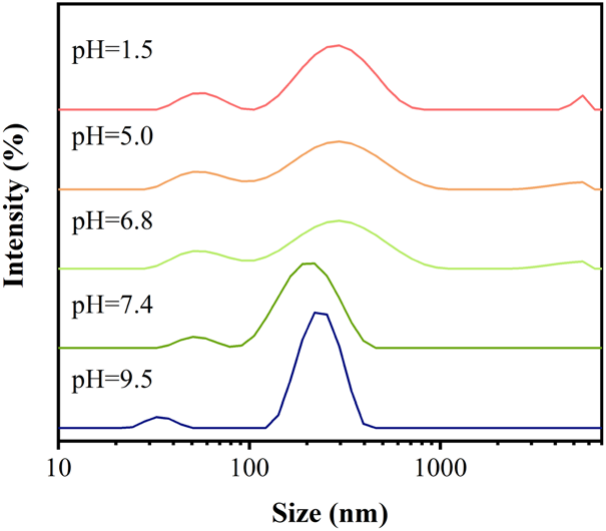
Particle size distribution of nanoparticles in different pH media.

In Fig. 4, the nanoparticles showed similar peak particle size and particle size distribution in neutral and alkaline environments. While incubated in acidic environment, the particle size became larger, the distribution gradually broadened and a strong absorption peak appeared at 5500 nm, which was attributed to the fact that one of the DAO, as a derivative of DA, had similar properties with DA and tended to dissolve under acidic conditions,^26^ in addition, the Schiff base structure in PDAO and FA-DAO was broken under acidic environment,^27^ and these factors resulted in the nanoparticles' particle size gradually enlargement and even disintegration due to structural damage. This phenomenon is conducive to drug-carrying nanoparticles cleaving and releasing drugs in the acidic environment of the tumour, and exerting their passive targeting ability of enriching drugs at the tumour site.

### Examination of nanoparticle drug-carrying capacity

**Fig. 5:**
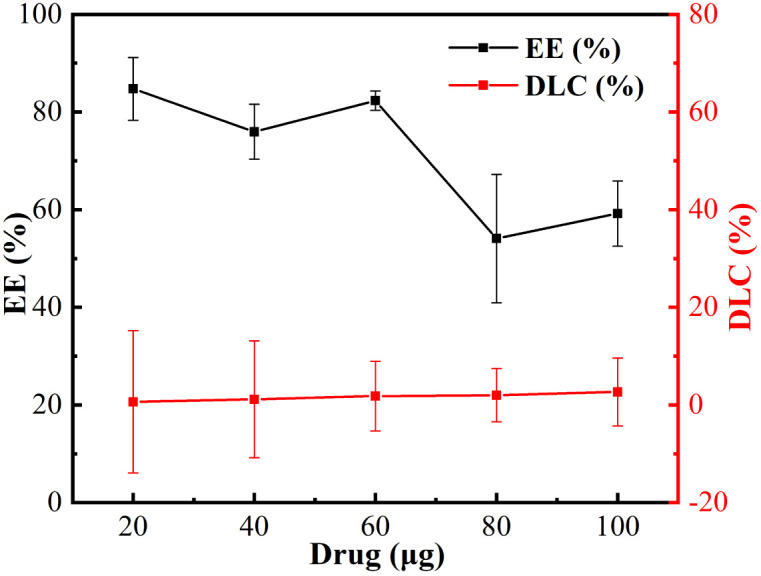
EE and DLC of nanoparticles.

Curcumin (Cur), as a drug with some antitumour activity,^28^ has been concerned widespread and studied as a drug model high frequency for its water insolubility. In this study, Cur was used as a hydrophobic antitumour drug model to evaluate the drug-carrying capacity of the nanoparticle, as shown in the Fig. 5, it can be seen that with the increase of the dosage, the Drug Loading Capacity (DLC) slowly increases, but the Entrapment Efficiency (EE) decreases, this is because the addition of too much Cur leads to a decreasing of EE of this drug-carrying nanoparticle, which may be due to the fact that the DLC of this nanoparticle is limited, and when the system cannot be loaded with too much Cur, the excess Cur will be adsorbed on the surface of the nanoparticle or dispersed in aqueous solution, which is sunk by centrifugation leading to a reduced EE.^29^ In order to achieve the conditions that the DLC is larger and the EE is over than 80%, we chose the dosage of 60 μg as the optimal condition. At a dosage of 60 μg, the EE and drug loading capacity DLC were close to 80% and 3%, respectively, indicating that the nanoparticles possessed a good ability to load hydrophobic drugs.

### Release behaviour of drug-loaded nanoparticles

**Fig. 6:**
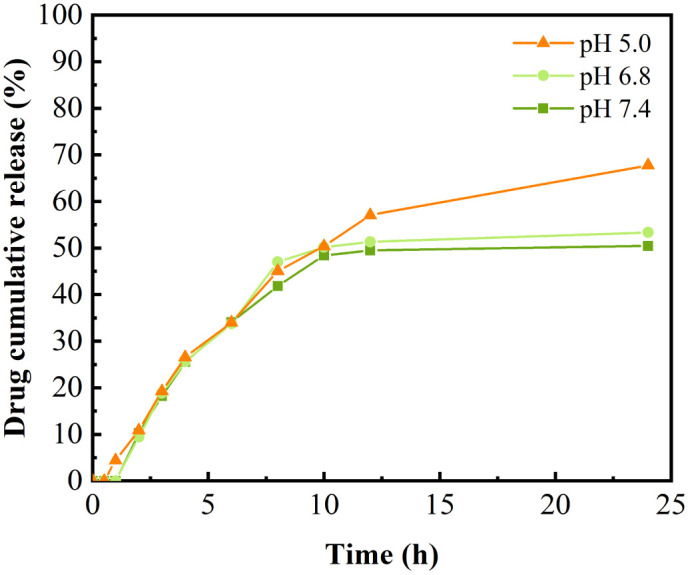
Release profiles of drug-loaded nanoparticles in different release media.

In the Fig. 6 the drug-carrying nanoparticles could be released continuously within 24 h. The cumulative release rate at 24 h was 70% and 50% in acidic environments of pH 5.0 and pH 6.8, respectively, which was significantly higher than that in pH 7.4. This was attributed to the fracture of the Schiff base structure due to its instability in acidic environments and the dissolution of DAO to some extent in acidic environments, which synergistically resulted in the decrease of the stability of the drug-carrying nanoparticles and structural disruption, thus releasing the drug. This suggests that the nanoparticles have some slow-release and pH-responsive ability, which can be used for the continuous treatment of tumours to improve the anti-tumour effect of hydrophobic drugs and reduce the frequency of administration.

### Cytotoxicity assay MTT

**Fig. 7:**
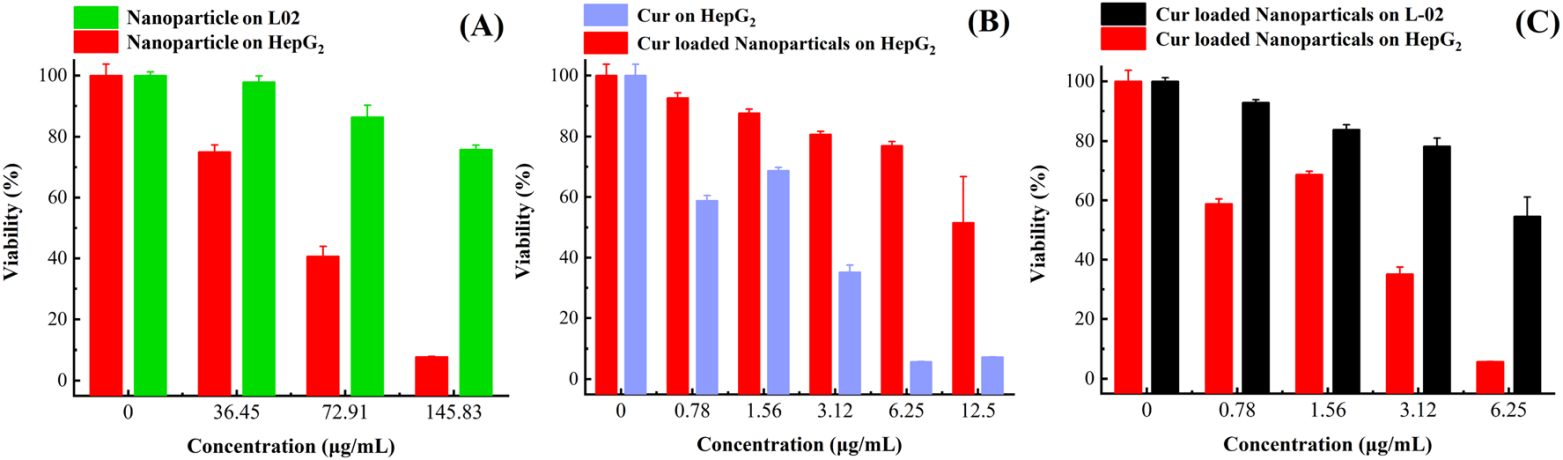
(A) Effect of blank nanoparticles on HepG2 and L02 cells. (B) Effect of API Cur and drug-loaded nanoparticles on HepG2 cells. (C) Effect of drug-loaded nanoparticles on HepG2 and L02 cells.

In order to verify the biosafety of this nanocarrier, the ability of targeting the tumour cells and the anti-tumour ability of drug-loaded nanoparticles, a series of experiments were designed in this study by selecting normal cells L02 and cancer cells HepG2.^30^ As shown in Fig. 7(A), the blank nanocarrier had no effect on normal cells in the concentration range of 0–145 μg mL^−1^, and had a good safety; surprisingly, the blank nanocarrier had a certain inhibitory effect on tumour cells; as shown in Fig. 7(B), the nanocarrier made the anti-tumour effect of Cur nearly more than double at the Cur concentration of 3.12 μg mL^−1^ in both cases; and as shown in Fig. 7(C). At Cur concentrations of 0–6.25 μg mL^−1^, the nanoparticles had less effect on normal cells, whereas they had a significant inhibitory effect on tumour cells. This is because the nanocarrier is more likely to be actively ingested by tumour cells due to the modification of FA;^31^ at the same time, the tumour inhibitory effect at a lower dose is due to the acidic microenvironment of tumour cells, and the structural instability of the Schiff base in the nanocarrier in the acidic condition and the fracture of the nanocarrier prompts nanoparticles to disassemble to release more drugs. It showed higher antitumor effect at lower drug dose. In conclusion, the nanocarriers prepared in this paper are able to improve the antitumor effect of hydrophobic antitumour drugs through active-passive targeting by linking the functional molecules such as PEG and FA to DAO through the Schiff base structure with a certain design.

## Conclusions

In summary, we successfully synthesised a series of functional molecules such as DAO and PDAO with Schiff base structure and FA-DAO, and prepared the functional nanoparticles by compounding them. The nanoparticle size was 146.53 ± 16.50 nm, and the structure of the nanoparticles was changed under acidic conditions, they exhibited continuous release for 24 hours, with a cumulative release rate reaching nearly 70%. Furthermore, the drug-loaded nanoparticles have the ability to passively target tumours based on pH response and actively target tumours based on FA, which can effectively improve the antitumour effect of Cur. This work provides new insights into the construction of dopamine-based tumour-targeting delivery systems and also promising to be a good delivery platform for hydrophobic anticancer drugs.

## Materials and methods

### Materials

Curcumin (Cur), linoleic acid and folic acid (FA) were purchased from Shanghai Macklin Biochemical Technology Co., Ltd, acetonitrile was acquired from Merck KGaA methanol pyrene, 1-ethyl-3-(3-dimethylaminopropyl) carbodiimide hydrochloride (EDC-HCl), *N*-hydroxybenzotrizole (HOBt) and *N*,*N*-dimethylformamide (DMF) were purchased from Shanghai Acmec Biochemical Technology Co., Ltd, dopamine hydrochloride (DA-HCl) was obtained from MERYER, mPEG-NH_2_ from Ponsure Biological, Shanghai.

### Synthesis of DAO, PDAO and FA-DAO

Slight modifications were made to the present method.^32^ 1 mmol each of linoleic acid, 1-ethyl-(3-dimethylaminopropyl) carbodiimide hydrochloride and 1-hydroxybenzotriazole were added to *N*,*N*-dimethylformamide, followed by stirring for 40 min in an ice-water bath at 0 °C, then stirring for 120 min at room temperature, and then 1 mmol and 3 mmol of dopamine hydrochloride and triethylamine were added, followed by stirring for 14 h at room temperature; finally, the dopamine derivative DAO was purified by fast column chromatography; then 0.11 mmol of mPEG-NH_2_ and 0.1 mmol of DAO were dissolved in ethanol solution of sodium hydroxide (4 × 10–4 g L^−1^) and stirred at room temperature for 24 h. At the end of the reaction, the solvent was removed by rotary evaporation, and dissolved in ethyl acetate, and then the product was purified by SPE column: activation with petroleum ether, elution with lysis buffer (ethyl acetate : methanol : cyclohexane = 10 : 1 : 1), and elution with eluent (ethyl acetate : methanol : cyclohexane = 1 : 10 : 1). The eluent was eluted with ethyl acetate : methanol : cyclohexane = 1 : 10 : 1, the eluent was collected and rotary evaporated to obtain red to reddish brown oil PDAO; finally, 0.11 mmol of FA and 0.1 mmol of DAO were dissolved in DMSO, 0.01 mmol of triethylamine was added, stirred at room temperature for 24 h, lyophilised for 24 h, ethanol was added to precipitate FA, and the supernatant was taken and rotary evaporated to give the light-yellow oil FA-DAO.

### Preparation of nanoparticles

PDAO, DAO and FA-DAO were dissolved in methanol to prepare stock solution with a concentration of 1 mg mL^−1^, and a certain amount of each stock solution was taken and mixed, vortexed for 30 s, ultrasonicated for 10 min, the solvent was removed by rotary evaporation, and the solvent was added to the water and vortexed for 30 s, and then heated at 50 °C for 30 min, and ultrasonicated for 10 min, and the nanoparticles were obtained.

### Preparation of Cur-loaded nanoparticles

Dissolve Cur in methanol to prepare a stock solution with a concentration of 1 mg mL^−1^, take a certain amount of each stock solution of the above PDAO, DAO and FA-DAO and mix it (PDAO : DAO : FA-DAO : Cur = 1 : 0.3 : 0.1 : 0.02), vortex for 30 s, ultrasonicated for 10 min, rotary evaporation to remove the solvent, add water to vortex for 30 s, and then heat it up at 50 °C for 30 min. The Cur-loaded nanoparticles were obtained by ultrasonication for 10 min, and the solvent was removed by rotary evaporation.

### Characterization

#### Structural characterization of DAO, PDAO and FA-DAO

The structure of DAO, PDAO and FA-DAO were characterized by Fourier transform infrared spectroscopy (FTIR). The samples were homogeneously applied to the detection stage and analysed in an infrared spectrometer (Nicolet iS50 FT-IR Spectrometer, Thermo Scientific, USA), acquiring scans at 500–4000 cm^−1^ from which the IR absorption peaks were recorded.

In order to obtain more precise structure information, we analysed the above three substances by hydrogen nuclear magnetic resonance (^1^H-NMR). A total of 30 mg of the samples were dissolved in DMSO-d_6_ separately and loaded into a hydrogen NMR spectrometer (AVANCE III HD 400, Bruker, Germany), and then the hydrogen spectra were recorded.

#### Characterization of nanoparticles size

The above nanoparticle stock solutions were taken and incubated in water solution and the particle size was measured by a laser nano-particle size analyser (ZetasizerNanoZS90, Malvern, UK). Instrument parameters were set to: detector position: 90°; temperature: 25 °C; PEG refraction rate: 1.45; refractive index of water solution: 1.33.

#### Transmission electron microscopy (TEM, Talos F200×, FEI, USA)

Configure 0.3 mg mL^−1^ aqueous solution of drug-carrying nanoparticles, dilute 100 times, pipette one drop onto the ultra-thin carbon-supported membrane, waiting for 15 min, then stain with 1% aqueous phosphotungstic acid solution, and dry under infrared light. The morphology was subsequently observed under the TEM.

#### Determination of nanoparticle CAC and UV spectrum

The above stock solutions were mixed in a certain amount (PDAO : DAO : FA-DAO = 1 : 0.3 : 0.1), methanol was removed by rotary evaporation, 5 mL water was added, heated at 50 °C for 30 min, and ultrasonicated for 10 min to obtain the nanoparticle stock solutions. Subsequently, they were diluted 10 times and 100 times respectively. Then 0.1, 0.2, 0.5 and 0.8 mL of the solution were taken from the nanoparticle stock solutions, diluted 10 times and diluted 100 times respectively to the headspace bottle containing 0.12 μg of pyrene, and then water was added to the obtained 12 bottles to 10 mL. The 12 different concentrations of the solution were heated for 1 h and ultrasonicated for 10 min. After cooling to room temperature, the fluorescence intensity was measured by fluorescence spectrophotometer (Lumina, Thermo Scientific, USA). The fluorescence scanning conditions were as follows: temperature 25 °C, fluorescence scanning excitation wavelength 335 nm, emission spectral range 350–450 nm, excitation slit set at 5.0 nm, and emission slit set at 2.5 nm.

Prepare aqueous solutions of different nanoparticles with the same concentration, and then measure their UV spectra at 250–600 nm (UV-Vis spectroscopy, Hitachi, Japan).

#### Determination of nanoparticle size in different pH media

The above nanoparticle stock solutions were taken and incubated in hydrochloric acid solution at pH 1.5, PBS buffer solution at pH 5.0, pH 6.8, pH 7.4 and pH 9.5 for 24 h, after which the particle size was measured by a laser nano-particle size analyser (ZetasizerNanoZS90, Malvern, UK). Instrument parameters were set to: detector position: 90°; temperature: 25 °C; PEG refraction rate: 1.45; refractive index of water solution: 1.33.

#### Entrapment efficiency and drug loading capacity

The entrapment efficiency (EE) and drug loading capacity (DLC) of the solution of Cur-loaded Nanoparticles were quantified by high performance liquid chromatography (HPLC). 5 mL of Cur-loaded nanoparticles aqueous solution was configured and 1.0 mL of Cur-loaded nanoparticles solution was accurately extracted and dissolved in 4.0 mL of methanol and mixed well. The remaining 4.0 mL of Cur-loaded nanoparticles solution was centrifuged at 7000 rpm min^−1^ for 20 min, and 1.0 mL of the supernatant was taken and added to 4.0 mL of methanol and mixed well. The peak areas of the nanoparticle solutions containing different amounts of Cur were measured by High Performance Liquid Chromatography (HPLC, ultimate 3000, Thermo Scientific, USA) (YMC-Pack ODS-AQ, 250 × 4.6 mm L. D. S-5 μm, 12 nm) (the mobile phase was composed of acetonitrile and 4% acetic acid solution ‘50 : 50, v/v’ at a flow rate of 1.0 mL min^−1^. The injection volume was 10 μL and the detection wavelength was 450 nm), and the actual content of Cur dissolved in the nanoparticle solution was calculated by standard curve, which was then brought into formula (1) and (2) to calculate the EE of the drug and DLC.1
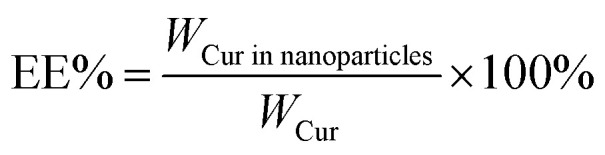
where *W*_Cur in nanoparticles_ denotes the drug content encapsulated in the nanoparticles and *W*_Cur_ denotes the amount of drug delivered.2
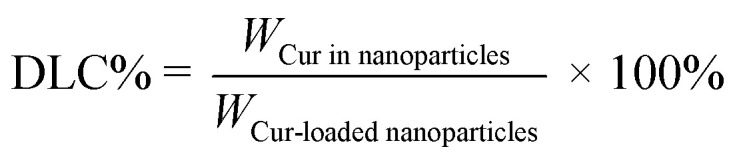
where *W*_Cur in nanoparticles_ denotes the drug content encapsulated in the nanoparticles, and *W*_Cur-loaded nanoparticles_ denotes the drug content in the nanoparticles and the total amount of carrier.

#### *In vitro* release assay

5 mL of Cur-loaded nanoparticles were packed into three 3500 MD dialysis bags of 10 cm length, tied at both ends, and at 37 °C for 24 h in conical flasks containing pH 5.0, pH 6.8 and pH 7.4 buffer solutions. At 0.5, 1, 2, 3, 4, 6, 8, 10, 12 and 24 h, 1 mL of the samples were taken and replenished with equal volumes of the same buffer solution, and the content was determined by the HPLC method. Cumulative release was calculated in accordance with formula (3)3
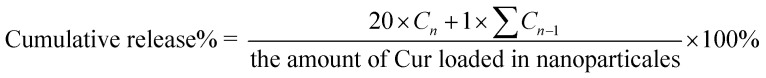


#### MTT experiment

L-02 or HepG2 cells in logarithmic growth phase were inoculated in 96-well plates (5 × 10^3^ cells per well), and the cells were incubated overnight in the cell culture incubator for adherent growth. Different sample concentrations were set, and the samples were added and incubated for 24 h. After incubation, 20 μL of MTT solution (5 mg mL^−1^) was added to each well and incubated for 4 h. The supernatant was discarded and 110 μL of DMSO was added, and the absorbance was measured at 490 nm (Varioskan™ LUX, Thermo Scientific, USA). The cell viability was calculated according to formula (4).4
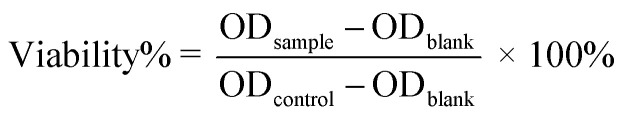


## Data availability

All data are replicated three times or more and are authentic and reliable, and can be obtained from the authors if needed.

## Conflicts of interest

There are no conflicts to declare.
